# Quantitative Analysis of the Antiepileptogenic Effects of Low Frequency Stimulation Applied Prior or After Kindling Stimulation in Rats

**DOI:** 10.3389/fphys.2018.00711

**Published:** 2018-06-18

**Authors:** Mostafa Jalilifar, Ali Yadollahpour, Ahmad Ali Moazedi, Zohreh Ghotbeddin

**Affiliations:** ^1^Department of Medical Physics, Faculty of Medicine, Ahvaz Jundishapur University of Medical Sciences, Ahvaz, Iran; ^2^Department of Biology, Faculty of Science, Shahid Chamran University of Ahvaz, Ahvaz, Iran; ^3^Department of Physiology, Faculty of Veterinary Medicine, Shahid Chamran University of Ahvaz, Ahvaz, Iran

**Keywords:** kindling, low frequency stimulation, spectral power, extracellular EEG, quantitative assessment

## Abstract

**Background and Objective:** Developing quantitative measures based on spectral analysis of electroencephalograph (EEG) recordings of neural activities plays an important role in developing efficient treatments for epilepsy. Such biomarkers can be used for developing open or closed loop approaches for seizure prediction or prevention. This study aims to quantitatively evaluate antiepileptogenic effects of low frequency stimulation (LFS) applied immediately before or after kindling stimulations using spectral power analysis of extracellular EEG in rat.

**Methods:** Nineteen adult rats were used: seven for kindle, six for LFS+Kindle (LFSK) and six for Kindle+LFS (KLFS). Four packages of LFS (1Hz) were applied immediately before or after rapid kindling stimulations. The power spectral densities of afterdischarge (AD) sections of EEG corresponding to different stages of kindling for delta (0–4 Hz), theta (4–8 Hz), alpha (8–12 Hz), beta (12–28 Hz), gamma (28–40 Hz) sub-bands, and theta/alpha ratio were comparatively investigated. Moreover, correlation between AD duration (ADD) and its different frequency components was calculated.

**Results:** Both LFSK and KLFS significantly increased delta and reduced beta and gamma oscillations, compared with kindle group. However, just the reduction in LFSK group was significant. Both protocols increased theta/alpha ratio, but just LFSK showed significant increase (*p* < 0.05). Although LFSK enhanced theta/alpha ratio more than KLFS, the difference was not statistically significant. Furthermore, strong correlation between each frequency sub band and ADD was not observed in kindle and LFS treated groups (both LFSK and KLFS).

**Conclusion:** Although behavioral assessments showed relatively the same level of antiepileptogenic effects for KLFS and LFSK, quantitative assessments showed more significant differences in the quantitative measures between the two protocols. Developing more quantitative EEG based measures correlated with LFS-induced effects can facilitate developing open or closed loop seizure prevention modalities.

## Introduction

Epilepsy is a chronic neurological disorder with 1.5% world population prevalence (Demos, 2005). It is characterized by recurrent seizures which disruptively propagate from the seizure origin to other regions of the brain. Cortical excitability of different areas of the brain is increased in epileptic patients (Lerchl et al., 1990; de Boer et al., 2008). Temporal lobe epilepsy (TLE) is the most common type of epilepsy in adults that originates in medial or lateral temporal lobe and spreads rapidly to other regions (Pritchard, 1986; Devinsky, 1991). Electrical kindling is a reliable experimental model to study TLE where repetitive electrical stimulations with a threshold intensity at particular sites of the brain induce progressive, generalized seizures through long term potentiation (LTP) mechanism (Goddard et al., 1969; Lothman et al., 1985). During the kindling process, afterdischarge (AD) waves appear in the electroencephalogram (EEG) baseline. ADs are electroencephalographic responses to seizures and they stem from the collective activity of neurons and produce consecutive large spikes in the baseline of EEG signals (Peterson and Albertson, 1982).

Animal studies have demonstrated that electrical stimulation of the epileptic focus, particularly in low frequency stimulation (LFS) may be an appropriate alternative treatment for intractable epilepsy (Velìšek, Velìšková and Stanton, 2002; Ozen et al., 2008; Jalilifar et al., 2017a). LFS can inhibit epileptic seizures through increasing the threshold for evoking neuronal action potentials (Albensi et al., 2004; Schrader et al., 2006). Moreover, administration of LFS especially in hippocampus and amygdala induces long term depression (LTD) as well as prevents kindling- induced LTP (Fujii et al., 1999; Albensi et al., 2004). Studies are ongoing to determine effective protocols of LFS for seizure inhibition (Shahpari et al., 2012). Time of LFS administrations is one of the main factors influencing the amount of antiepileptic effects of LSF. The behavioral data of our previous study showed that LFS application either before or after termination of kindling stimulation significantly increased AD threshold, inhibited kindling acquisition, and also increased the number of stimulations required to achieve kindling stages, compared with the control animals that received only kindling stimulation. Although administration of LFS prior to kindling stimulation produced a more inhibitory effect than the post kindling protocol, the difference was not statistically significant. To determine effective LFS parameters, efficient biomarkers should be developed for quantitative assessments of epileptogenesis process as well as the LFS induced effects. Most of the studies on kindling have used the behavioral assessments based on kindling stages and duration of AD to evaluate the LFS efficacy. However, behavioral assessments suffer subjective and objective errors since the identification of start and end of each stage, its duration, as well as duration of AD are determined manually. EEG signals have a high temporal and good spatial resolution making them appropriate measures to identify efficient LFS parameters as well as to determine the antiepileptic mechanisms of LFS (Yadollahpour and Jalilifar, 2014). However, visual assessments of EEG signals to identify and quantify the oscillatory activities and their behavioral correlates yield no valuable information. Using different spectral analysis methods of EEG enable researchers to identify, quantify, and characterize the oscillatory components in the EEG signals as well as to develop quantitative measures which are correlates of different behavioral features (Kleinfeld, 2008). The two main advantages of using spectral analysis in electrophysiological studies are determination of the number of degree of freedom for calculation of confidence of interval is more convenient in frequency domain than the time domain and two the most of biological phenomena have simpler representation in the frequency domain (Kleinfeld, 2008). In this regard, the goal of quantitative EEG is to identify different measures in frequency domain and consequently investigate the brain functions. Fourier analysis is a main group of spectral analyses where time series signals, namely EEG, are decomposed into sinusoidal functions.

This study focuses on the frequency domain features of the epileptic activities from the AD of the EEG signals that could provide more clear understanding of the intrinsic neural network involved in kindling process. Developing quantitative and objective assessments of LFS effects on epileptogenesis using EEG signals can reduce the objective and subjective errors present in the behavioral assessments. In our previous studies we determined the main spectral features of EEG signals in different stages of kindling and also assessed the variations of different EEG based measures during progression of kindling (Jalilifar et al., 2016, 2017b). Our findings along with the findings of previous studies showed significant correlations between variations of specific sub-bands of EEG with different phases of epileptogenesis: We classified EEG signal into different sub-bands whose powers are considered as a synchronization of neural discharge index. These sub-bands include delta (1–4 Hz), theta (4–8 Hz), alpha (8–12), beta (12–28 Hz) and gamma (28–40 Hz). Delta frequencies are synchronized in deep sleep state, associated with seizure-like activities in the brain (Walter, 1936). Theta waves are usually recorded from Medial Septum area of hippocampus and they are related with voluntary movements of rats. Alpha oscillations originate from occipital and other sensory areas. Several studies have demonstrated that alpha waves are affected by thalamus and sensorimotor cortex in rats (Hughes and Crunelli, 2005; Shaker, 2006). Beta and gamma rhythms are predominant in the neocortex and hippocampus of consciousness humans and animals (Haenschel et al., 2000). Theses waves are also augmented in generalization of epileptic seizures. Moreover, suppression of beta and gamma frequencies can inhibit the progression of epileptogenesis process (Tsuchiya and Kogure, 2011). In addition to the above sub-bands, alterations of theta/alpha ratio have shown a significant correlation with the level of alertness in cognitive studies (Sadighi Alvandi et al., 2015) because theta sub-bands are usually correlated with learning and alertness disorders, and they are often emerged with high amplitude in the epileptic patients while alpha frequencies are generally the most stable brain waves. In line with our previous studies, this study aims to quantitatively investigate the antiepileptogenic effects of LFS applied either immediately prior or after daily kindling stimulation in amygdale rapid kindling model in rats. In addition, the time dependent effects of LFS on kindling induced epileptogenesis are investigated using EEG spectral power analyses. To do so, the effects of LFS in the two protocols on the spectral powers of delta (1–4 Hz), theta (4–8 Hz), alpha (8–12 Hz), beta (12–28 Hz), gamma (28–40 Hz), and theta/alpha ratio are comparatively assessed.

## Materials and Methods

### Animals and Surgery

All of the experimental procedures of this study were approved by local ethics committee of AJUMS which were in complete accordance with the guide for the care and use of laboratory animals set by the National Academy of Sciences (National Institutes of Health Publication No. 86-23). Adult male Wistar rats (weighing 190–210 g at the time of surgery) were obtained from the animal house of Ahvaz Jundishapur University of Medical Sciences (AJUMS) (Ahvaz, Iran). They were accommodated individually in a colony room with an ambient temperature (25 ± 2°C) and artificial 12:12-h light-dark cycle (light on at 6.30–18.30).

The rats were anesthetized with intraperitoneal injection of the mixture of Ketamine (100 mg/kg) and Xylazine (10 mg/kg) (Esmaeilpour et al., 2013). and underwent surgery process: four holes were drilled stereotaxically on the skull two holes for anchor screws, one for implantation of a monopolar stainless steel electrode used as ground and another for placement of a tripolar stainless steel electrode (two poles for stimulation and one for recording) in the right amygdala according to the Paxinos and Watson atlas coordinates: anteroposterior: -2.5 mm; lateral: 4.8 mm; vertical: 7.2 and 0.2 mm below the skull (Paxinos et al., 2009). After the placement in the coordinates, the electrodes were attached into a socket and maintained with dental acrylic cement.

### Kindling and LFS

**FIGURE 1:**
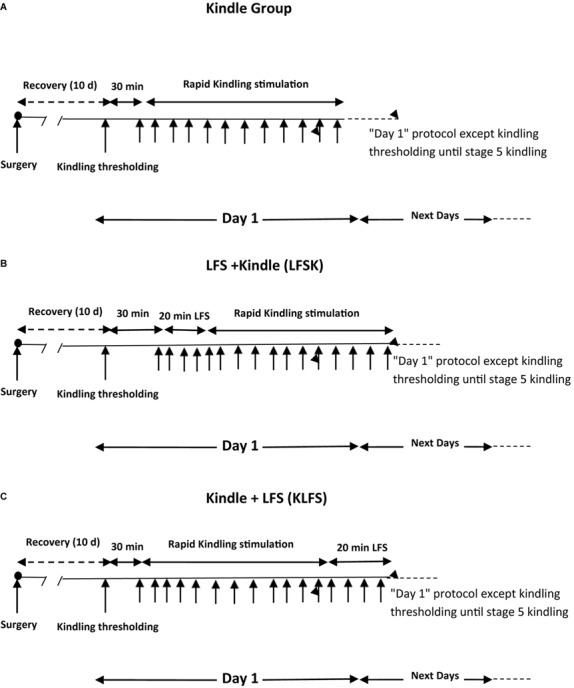
Schematic diagram of experimental groups. **(A)** Kindle group. **(B)** LFS + kindle (LFSK) group, **(C)** kindle + LFS (KLFS) group.

Nineteen rats were randomly divided into 3 groups including one kindle (*n* = 7) as control group and two treatment groups of LFS + kindle (LFSK) (*n* = 6) and kindle + LFS (KLFS) (*n* = 6). Following a 10-day recovery period after surgery, the threshold intensity for kindling stimulation was determined as the minimum intensity required to evoke at least 6 s ADs with the amplitude of at least 2.5 times higher than the baseline EEG (Yadollahpour et al., 2014). It was determined by a 3 s of monophasic square wave of 50 Hz applied initially at 30 μA and increased in increment of 15 μA at minimum of 30 min intervals. The rats that represented no AD with maximum 350 μA current intensity were excluded from the experiment. Moreover, the animals that entered in deep inhibitory state and those animals showed unusual response including severe reactions at the kindling threshold intensity were excluded from the study. All rats were subjected to kindling stimulation consisting of a 3 s of monophasic square wave (1 ms duration) of 50 Hz with the threshold intensity applied 12 times per day at 5 min intervals (Shahpari et al., 2012). The kindling stages for the further assessments were recorded by the researchers based on the following behavioral characteristics: stage 1 characterized by mouth and facial movements, stage 2 by head nodding, stage 3 by Forelimb clonus, stage 4 by rearing, and stage 5 characterized by falling and loss of balance (Racine, 1972). Kindling stimulations in the kindle group were continued until the observation of the stage 5 of kindling. The average days in the control group to reach the stage 5 was 4.42 ± 0.53 days. Therefore, to compare the behavioral data between the control and LFSK and KLFS groups on a standard basis, the animals of these groups were stimulated for 5 days. In the LFSK group, four packages of LFS with 5 min interval were applied immediately before the start of 12 daily kindling stimulations, while in the KLFS group these packages were applied immediately after termination of 12 daily kindling stimulations (**Figure 1**). Each LFS package consisted of 200 monophasic square pulses, 0.1 ms pulse duration at 1 Hz with the threshold intensity (Yadollahpour et al., 2014). After the completion of the experiments the animals were sacrificed using Co2 in a euthanizing chamber. All efforts were made to minimize animal suffering and reduce the number of animals used in this study.

### Spectral Analysis of afterdischarge

Electroencephalograph signals were recorded through the electrode implanted in the amygdale and monitored with the Electromodule system (ScienceBeam Co, Iran). Data were digitized at a sampling rate of 10 KHz. During the kindling acquisition, the time and duration of each kindling stage were saved as an event file which should be considered in extracting each stage. It should be noted that only the AD parts of EEG were selected and treated with Hann window function with an overlap of 50% and then were transferred into the frequency function by Fast Fourier Transform (FFT) and their power spectrum and the power of each sub band including delta (1–4 Hz), theta (4–8 Hz), alpha (8–12 Hz), beta (12–28 Hz), gamma (28–40 Hz), and theta/alpha ratio were determined using MATLAB version 2013b for Windows.

### Statistical Analysis

Data were represented as the mean ± standard error of mean (SEM). The normality of the data was evaluated using Kolmogorov–Smirnov test. A one-way analysis of variance (ANOVA) following Bonferroni’s *post hoc* was used to compare different sub bands power of baseline periods between the experimental groups. A two-way ANOVA following a *post hoc* Bonferroni’s test was performed to compare different sub bands power of AD related to Racine stages and also theta/alpha ratio of EEG between kindle (*n* = 7), LFSK (*n* = 6), and KLFS (*n* = 6) groups. The correlation between AD duration (ADD) and the spectral powers of different EEG sub bands were assessed using Pearson’s correlation coefficient. All Statistical analyses were performed with IBM SPSS 21 for windows. For all analyses, the tests were carried out two-sided and significance was set at *p* < 0.05.

## Results

### Kindling Induced EEG Features

In the recently published papers, we identified the main quantitative features of different kindling stages during epileptogenesis (Jalilifar et al., 2016) and then for the main phases of seizure acquisition initial, localized and generalized seizure stages in the amygdale rapid kindling model (Jalilifar et al., 2017b) (detailed data not provided here). The main features of kindling were as follows: The kindling acquisition process was accompanied by increase in delta (1–4 Hz) and theta (4–8 Hz) waves in the stages of 3, 4, and 5, compared with the control group. Moreover, with the progression of the kindling process, high beta (20–28 Hz) and gamma (28–40 Hz) oscillations were reduced. Delta sub-band power significantly increased during generalized seizure stages (GSSs) (stages 4 and 5). Furthermore, the theta/alpha ratio in the localized seizure stage (stage 3) (LSS) was higher than GSSs and the sham group.

### General EEG Features of the Experimental Groups

Afterdischarge duration was significantly decreased following application of LFS either before or after the kindling stimulation as compared with the kindle group (*F*[2,78] = 19.682, *p <* 0.05) (Jalilifar et al., 2017b). LFS could also significantly prevent the generalization of behavioral stages during the kindling procedure in a way that all animals in the kindle group represented GSSs of the kindling process within 5 stimulation days while only one animal in the LFSK group (16%) and two animals (32%) in the KLFS showed GSSs at the end of the experiment. The animals in both LFSK and KLFS groups received higher numbers of numbers to exhibit LSS and GSSs of the kindling in comparison with the kindle group (for stage 2: *H*[2] = 6.725*, p* > 0.05, stage 3: *H*[2] = 8.498, *p* < 0.05, stage 4–5: *H*[2] = 13.658, *p* < 0.05). Animals in the LFSK group showed LSS and GSSs of the kindling process with higher numbers of stimulation than the KLFS; however, the difference was not significant (*p* > 0.05) (Jalilifar et al., 2017a). Moreover, the daily stages represented in the LFSK and KLFS groups were significantly decreased compared with the kindle group (for day 2: *H*[2] = 10.191, *p* < 0.05, day 3: *H*[2] = 13.696, *p* < 0.05, day 4: *H*[2] = 12.003, *p* < 0.05, day 5: *H*[2] = 10.667, *p* < 0.05) (Jalilifar et al., 2017a).

Animals in the experimental groups showed different number of main phases of seizures. However, in the kindle group all animals achieved 61 times initial seizure stages (stages 1 and 2) (ISSs), 42 LSS, and 35 times GSSs, whereas the LFSK animals showed 38 times ISSs, 13 LSS, and 4 times GSSs. Besides, the KLFS rats showed 39, 14, and 6 times ISSs, LSS, and GSSs, respectively which were registered for further analyses (Jalilifar et al., 2017a).

**FIGURE 2:**
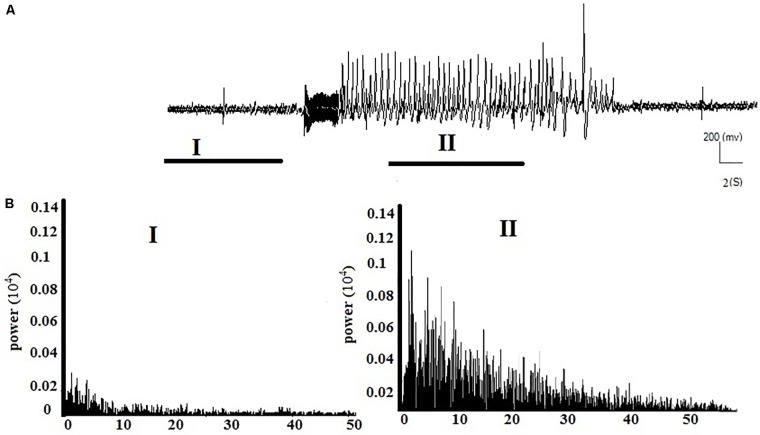
Example of the EEG record and corresponding FFT analysis in the kindle group. **(A)** A continuous baseline EEG record from the baseline period (I) to the AD duration (II). **(B)** The power spectrum corresponding to each phase of the EEG using FFT analysis.

**FIGURE 3:**
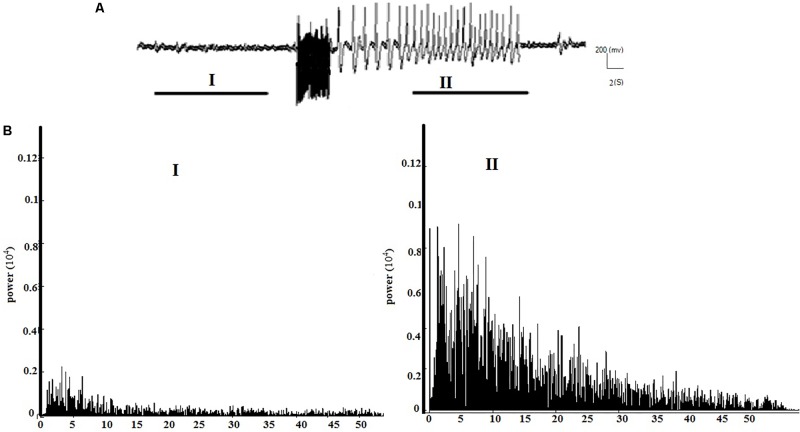
The spectral powers of the baseline and AD section of EEGs and the corresponding FFT analysis in the LFSK group. The LFSK and KLFS groups showed no significant differences in spectral powers. **(A)** A continuous baseline EEG (I) to the AD section EEG (II). **(B)** The spectral powers corresponding to baseline EEG and epileptic activities of the EEG signals.

**Figures 2A,B** respectively show the baseline and the epileptic (AD section) EEG records in the kindle group. **Figures 3A,B** respectively show examples of baseline and AD section EEGs in the LFS treated group. We recorded a 7 s length EEG of baseline neural activities prior to kindling stimulation and performed the FFT analyses on the 7 s length EEGs of baseline and epileptic activities in different groups. **Figure 3** represents a baseline EEG and epileptic EEG in the LFSK group (**Figure 3**). The FFT analyses for the baseline and epileptic activities of the LFSK and KLFS did not significantly differ; therefore, we presented one example from the LFSK. In the all experimental groups, delta and theta components [Low Frequency Band (LFB) (0–8 Hz)] were dominant bands during baseline activity, while Mid Frequency Band (8–12 Hz) (MFB) and High Frequency Band (12–40) (HFB) activities were enhanced along with the spreading epileptic activities (emerging AD waves). Moreover, during the AD period, larger spikes occurred at higher frequencies in the power spectrum of the EEG, compared with the baseline periods (**Figures 2**, **3**).

**FIGURE 4:**
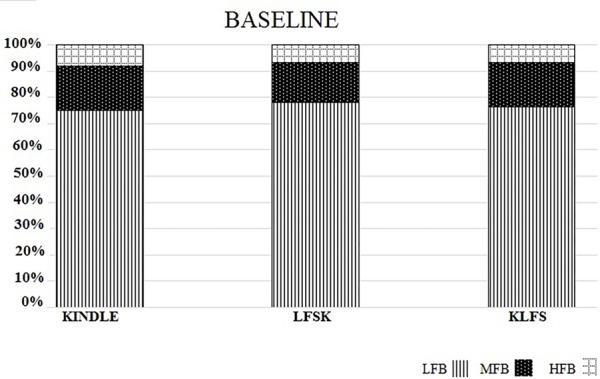
The spectral power density percentage of the three experimental groups for the baseline activities. There was no significant difference in the low (LFB), mid (MFB), and high frequency band (HFB) powers between the experimental groups (*p* > 0.05).

The power spectral density percentages of LFB, MFB, and HFB of the baseline EEG were compared between the three experimental groups. A one-way ANOVA test showed no significant difference in the LFB power [*F*(2,16) = 1.778, *p* > 0.05], MFB [*F*(2,16) = 2.053, *p* > 0.05], and HFB [*F*(2,16) = 1.372, *p* > 0.05] between the Kindle, LFSK and KLFS groups (**Figure 4**).

### Spectral Analysis of AD

**FIGURE 5:**
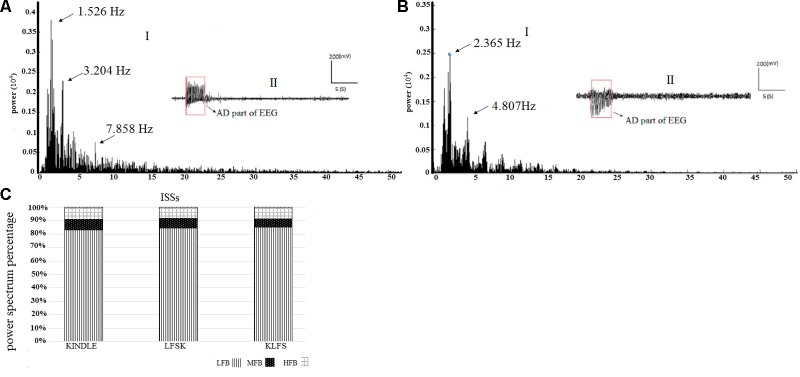
**(A-I)** An example of EEG power spectrum and **(A-II)** corresponding EEG signal of the ISS in the kindle group. Spectral power of only AD section or epileptic waves is performed. **(B-I)** The power spectrum of the ISS in the LFSK group. The LFSK and KLFS groups showed no significant differences in spectral powers. **(B-II)** An example EEG signal of ISS in the LFSK group. There was no significant difference between the control and intervention groups. **(C)** The power spectrum density percentage of the ISS sections of the EEG signals in the experimental groups. There was no significant difference in the power of different sub-bands between all groups (*p* > 0.05).

To clarify the effect of LFS on the frequency components of AD, the power spectrums of the kindling stages were compared between the experimental groups. **Figures 5A,B** are representative EEG signals of ISSs of the kindling acquisition in the kindle and also in the LFSK or KLFS groups, respectively. Since the induced changes in the power spectrum of both the LFSK and the KLFS in ISSs were relatively similar, we only demonstrated an example of the LFSK in **Figure 5**. There was no significant difference in the power spectrum of ISSs between the kindle and LFS treated animals (**Figures 5A,B**). According to **Figure 5C**, the LFB was dominant in ISSs of all the experimental groups. However, there was no significant difference in the LFB [*F*(2,18) = 0.2, *p* > 0.05], MFB [*F*(2,18) = 0.613, *p* > 0.05], and HFB [*F*(2,18) = 0.164, *p* > 0.05] power between the experimental groups (**Table 1**). It should be noticed that the animals in the kindle group showed totally 61 times ISSs while the LFSK and KLFS animals represented totally 38 and 39 times ISSs respectively which were included in this part of the analysis.

**Table 1:** The raw values and descriptive statistics of the LFB, MFB, and HFB in ISSs between the experimental groups.

Stage	Group	Sub band	Mean	Standard deviation (*SD*)	Statistics	*P*-value
ISSs	LFB	Kindle	0.828	0.088	*F*(2,18) = 0.2	*P* = 0.821
		LFSK	0.845	0.043		
		KLFS	0.851	0.057		
	MFB	Kindle	0.082	0.041	*F*(2,18) = 0.613	*P* = 0.554
		LFSK	0.065	0.032		
		KLFS	0.065	0.019		
	HFB	Kindle	0.088	0.046	*F*(2,18) = 0.164	*P* = 0.851
		LFSK	0.081	0.035		
		KLFS	0.074	0.047		

*There was no significant difference in the power of different sub-bands between the experimental groups.*

**FIGURE 6:**
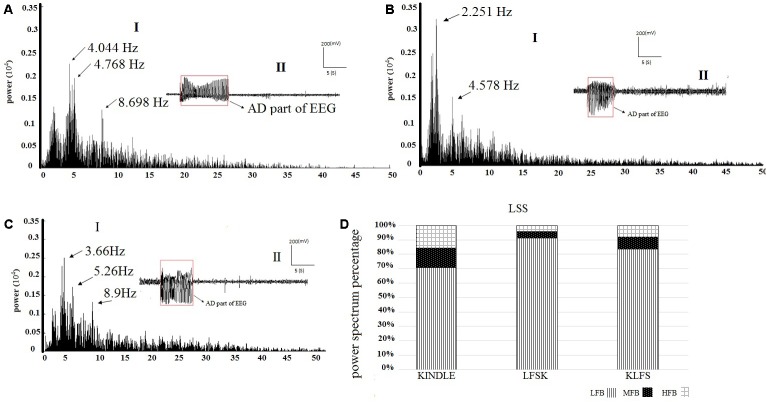
**(A-I)** An example of EEG power spectrum of the LSS and **(A-II)** corresponding EEG signal in the kindle group. The mid frequency (8–12 Hz) oscillations were evident in this stage. The peaks occurred at higher frequencies compared with ISSs of the kindle group. Furthermore, high frequency activities were more than ISSs of the kindle group. **(B-I)** The power spectrum of the LSS in the LFSK group. **(B-II)** An EEG signal example of LSS in the LFSK group. There were two peaks at 2.25 Hz and 4.58 Hz in the low frequency range (0–8 Hz). **(C-I)** The power spectrum of LSS in the KLFS. C-II: An EEG signal example of LSS in the KLFS group. **(D)** The power spectrum density percentage of LSS sections of the EEG signals in the experimental groups.

**Figures 6A–C** show examples of the power spectrum of LSS of the kindling acquisition in the kindle, LFSK, and KLFS groups, respectively. Application of LFS immediately either before or after the kindling stimulation reduced mid frequency (8–12 Hz) and high frequency (12–40 Hz) oscillations induced in LSS than the kindle group. We compared the power spectrum density percentages of LSS in the kindle, LFSK, and KLFS groups (**Figure 6D**). A One-Way ANOVA indicated a significant difference in the LFB power between the experimental groups [*F*(2,12) = 8.788, *p* < 0.05]. In this regard, application of the LFS either before or after the kindling stimulation significantly increased the LFB power, compared with the kindle group (*p* < 0.05).

In addition, there was a significant difference in the MFB [*F*(2,12) = 5.543, *p* < 0.05] and HFB [*F*(2,12) = 7.417, *p* < 0.05] power between the experimental groups (**Table 2**). In fact, MFB and HFB powers were significantly decreased in LSS of the LFSK, compared with the kindle group (*p* < 0.05). Although the MFB and HFB powers in the KLFS group were lower than the kindle, the difference was not significant (*p* > 0.05) (**Table 2**). Moreover, there was no significant difference in different sub-bands power of the EEG signals between the LFSK and KLFS groups (*p* > 0.05) (**Table 2**).

**Table 2:** The raw values and descriptive statistics of the LFB, MFB, and HFB in LSS between the experimental groups.

Stage	Group	Sub band	Mean	*SD*	Statistics	*P*-value
LSS	LFB	Kindle	0.7091	0.1005	*F*(2,12) = 8.788	
		LFSK	0.9124	0.0223		*P* = 0.014
		KLFS	0.8825	0.0465		*P* = 0.034
	MFB	Kindle	0.1323	0.0556	*F*(2,12) = 5.543	
		LFSK	0.0451	0.0058		*P* = 0.049
		KLFS	0.0582	0.0172		*P* = 0.1
	HFB	Kindle	0.1557	0.0595	*F*(2,12) = 7.417	
		LFSK	0.042	0.0219		*P* = 0.023
		KLFS	0.0592	0.0330		*P* = 0.053

*There was no significant difference in the power of different sub-bands between the experimental groups. The p-value represented the level of significance against the kindle group. LFSK and KLFS groups showed no significant difference in the power of different sub-bands in the LSS phase (p > 0.05).*

In the kindle group, the mid frequency (12–40 Hz) oscillations in LSS are more than the ISSs (**Figures 5A** and **6A**). Interestingly, not only did these figures confirm our result, but other power spectrum figures related to LSS of the experimental animals also supported the above idea and our results convinced us to only show one figure as a representative. It is worth noting that all animals in the kindle group demonstrated 42 times LSS which took on average 12.4285 s, and animals in the LFSK and KLFS showed 13 times LSS with an average of 9.373 s and 14 times with an average of 10.064 s, respectively.

**FIGURE 7:**
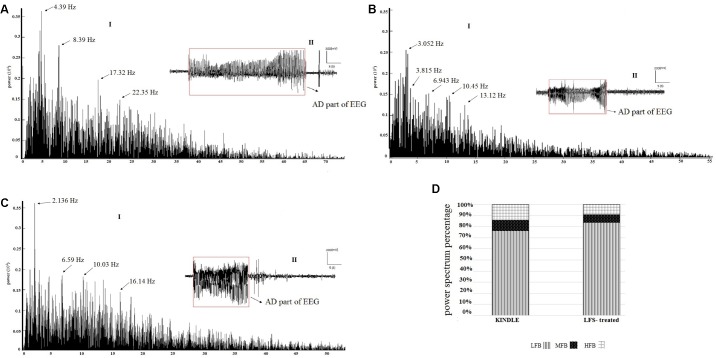
**(A-I)** An example of EEG power spectrum and **(A-II)** corresponding EEG signal of the GSSs in the kindle group. The mid (8–12 Hz) and high frequency (12–40 Hz) oscillations increased during epileptogenesis. **(B-I)** The power spectrum and **(B-II)** corresponding EEG signal of the GSSs in LFSK group. High frequency activities (12–40 Hz) were lower than the GSSs of the kindle group. **(C-I)** The power spectrum and **(C-II)** corresponding EEG of GSSs in the KLFS group. **(D)** The power spectrum density percentage of the GSSs in the kindle and intervention groups. Contribution of MFB and HFB in the LFS treated animals decreased.

**Figures 7A–C** are respectively examples of the power spectrum of the GSSs in the kindle and LFS treated groups. There were large peaks at mid (8–12 Hz) and high (12–40 Hz) frequencies in the kindle group (**Figure 7A**). Moreover, fewer peaks occurred at MFB and HFB in the GSSs in the LFSK and KLFS groups, compared with the Kindle (**Figures 7B,C**). The MFB and HFB components in the kindle group exceeded both LFS and KLFS groups (**Figures 7A–D**).

There were three peaks in the power spectrum of GSSs in the kindle group. These peaks were much larger and occurred at higher frequencies as compared with LSS of the kindle group. In addition, high frequency activities (12–40 Hz) were much higher than the LSS in the kindling group.

Only one rat in the LFSK and two rats in the KLFS group showed GSSs. Animals in the kindle group showed 35 times GSSs with an average length of 22.28 s, whereas animals in the LFSK and KLFS groups exhibited 4 and 6 times GSSs with length of 16.9 and 17.82 s, respectively. Therefore, to determine the power spectrum density percentage, we compiled these two groups into one LFS treated group (**Figure 7D**). Due to the imbalanced nature of the sample and fewer animals in both LFSK and KLFS groups showed GSSs than the kindle group, the statistical results were hardly reliable hence not reported here.

### Comparisons of ADD Frequency Components

**FIGURE 8:**
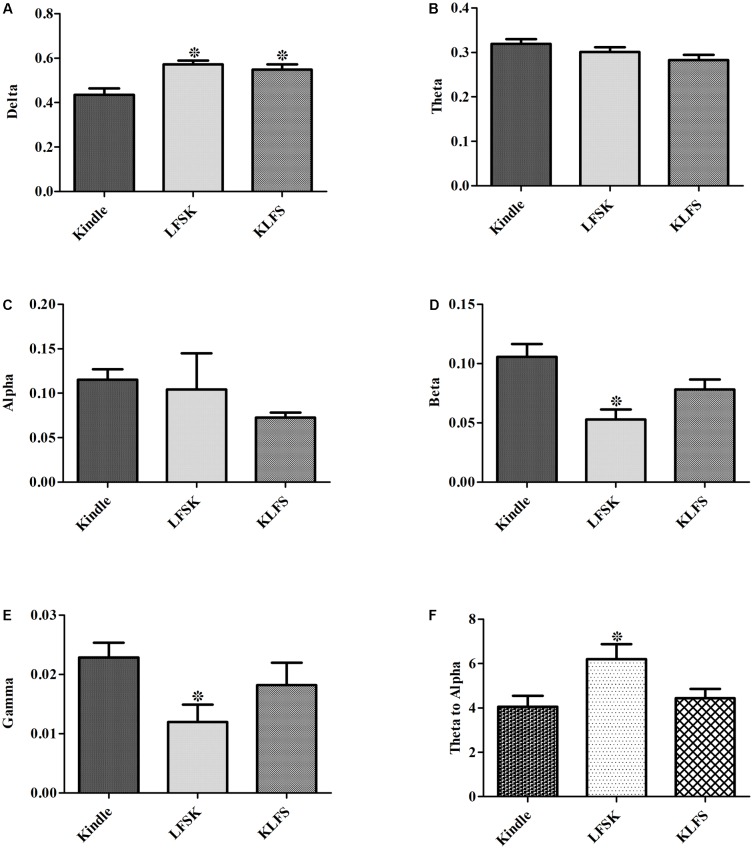
Contribution of each sub band to different groups. **(A–E)** Showed the power of Delta, Theta, Alpha, Beta, and Gamma for the kindle, LFSK, and KLFS, respectively. **(F)** Comparison of the Theta/alpha ratio in the experimental groups. The data were represented as Mean ± SEM. ^∗^: significant difference compared with kindle group (*P* < 0.05).

The power of different sub bands of EEG in the Racine stages was compared between different groups. A two-way ANOVA test showed a significant difference in the power of delta, beta and gamma sub bands between the groups (*p* < 0.05) (**Table 3**). A significant increase of delta power was observed in LFS treated groups compared with the kindle group (*p* < 0.05) (**Figure 8A**). However, there was no significant difference between the KLFS and LFSK groups (*p* > 0.05). In addition, administration of LFS only before kindling significantly reduced beta and gamma powers compared with the kindle group (*p* < 0.05), however these reductions were not significant in the KLFS group (**Figures 8D,E**). The LFSK protocol reduced the high frequency (12–40 Hz) power much more than the KLFS which likely lead to more inhibitory effects. The theta/alpha ratio increased in both LFSK and KLFS groups, compared with the kindle group; however, only the LFSK group was significantly different than the kindle group (*p* < 0.05). Moreover, the LFSK protocol increased the theta/alpha ratio more than KLFS protocol, whereas the difference was not statistically significant (*p* > 0.05).

**Table 3:** Results of a two-way ANOVA.

Sub band	*F*(2, 33)	*P*-value
Delta	5.974	0.006
Theta	1.261	0.297
Alpha	0.91	0.412
Beta	6.282	0.005
Gamma	4.373	0.021
Theta/Alpha	7.84	0.002

*According to the Table, there was a significant difference between the experimental groups in delta, beta, gamma power, and theta/alpha ratio (p < 0.05).*

### Comparison of Theta/Alpha Ratio

Due to the emergence of theta waves in the epileptic seizures and the association of alpha waves with alertness disturbances, in recent years the theta/alpha ratio has been considered as an important index to analyze the level of alertness as well as epileptic depolarization. In this regard, the theta/alpha ratio was evaluated in different stages of kindling and also it was compared between the kindle, the LFSK, and the KLFS groups. Seven animals in the kindle group, 6 in the LFSK, and 6 in the KLFS contributed to these analyses.

According to **Figure 8F**, application of LFS caused an increase of the theta/alpha ratio either before or after termination of kindling stimulation as compared with the Kindle group. However, the increase only in the LFSK group was significant (*p* < 0.05) (**Figure 8F**) (**Table 3**). Although application of LFSK could enhance theta/alpha ratio more than KLFS, the difference was not statistically significant.

### Correlation Between Frequency Contents and Duration of Epileptic Activities

The correlation analysis of ADD and powers of different EEG sub bands was determined. We aimed to quantitatively analyze the inhibition effect of LFS and only describe the difference between the Kindle and the LFS treated groups. Three rats in each LFSK and KLFS group showed LSS and one rat in the LFSK and 2 in the KLFS showed GSSs. Therefore, we combined the LFSK and KLFS animals into one LFS treated group to use the correlation test. To perform correlation analysis, EEG signals in each stage of Racine were divided into 3 sub bands including LFB (0–8 Hz), MFB (8–12), and HFB (12–40). We then determined the Pearson’s correlation coefficients between each of three sub bands and ADD for different kindling stages.

In the kindle group, no significant correlation observed between each frequency band and the different seizure stages. There was a low negative correlation between MFB and ADD in ISSs (*r* = -0.4, *p* > 0.05). In addition, a small correlation was observed between ADD and different frequency bands in GSSs (**Table 4**).

**Table 4:** Statistical data of the correlation test between EEG sub-bands and ADD in ISSs, LSS, and GSSs in the kindle group.

Stage		*R*	*P*-value
1&2 (ISSs)	ADD & LFB	0.38	0.4
	ADD & MFB	-4.0	0.364
	ADD & HFB	-233.0	0.466
3 (LSS)	ADD & LFB	-172.0	0.557
	ADD & MFB	0.2	0.667
	ADD & HFB	0.282	0.54
4&5 (GSSs)	ADD & LFB	-851.0	0.735
	ADD & MFB	0.168	0.718
	ADD & HFB	0.124	0.791

In the LFS treated animals, a significant or strong correlation was not observed between each frequency band and ADD in ISSs and LSS of the kindling. There was a low positive correlation between ADD and LFB in LSS (*r* = 0.427, *r* > 0.05) whereas a low negative correlation was witnessed between ADD and HFB (*r* = -0.448, *p* > 0.05) (**Table 5**). Since only three animals in the LFS treated groups developed GSSs, we cannot rely on the correlation test in GSSs in this group.

**Table 5:** Statistical data of the correlation test between EEG sub-bands and ADD in ISSs, LSSs, and GSSs in LFS treated groups.

Stage	LFSK-KLFS	*R*	*P*-value
1&2 (ISSs)	ADD & LFB	-123.0	0.536
	ADD & MFB	0.25	0.962
	ADD & HFB	0.4	0.432
3	ADD & LFB	0.427	0.399
	ADD & MFB	0.2	0.704
	ADD & HFB	-844.0	0.373
4&5 (GSSs)	ADD & LFB	0.721	0.488
	ADD & MFB	-557.0	0.456
	ADD & HFB	-927.0	0.48

## Discussion

The results of this study demonstrated that LFS administration either immediately before or after the kindling stimulation significantly inhibited kindling progression. Our behavioral results showed LFS immediately before kindling stimulation induced greater inhibition effects than the LFS applied immediately after kindling. However, the difference was not statistically significant. Wu et al. (2008) reported that therapeutic application of LFS was strongly time dependent in such a way that applying daily LFS immediately after kindling stimulations produced an antiepileptogenesis effect while delayed LFS applied after the cessation of AD not only could not retard kindling acquisition, but it also accelerated kindling progression (Wu et al., 2008). In addition, Sun et al. (2010) showed that LFS administration immediately after daily kindling stimulation could suppress the epileptogenesis process, whereas LFS administration before the termination of daily kindling stimulation, did not result in inhibitory effect (Sun et al., 2010). However, the findings of the previous studies on the time dependency of LFS effects were controversial necessitating further studies in this regard (Kile et al., 2010; Lucas et al., 2012; Shahpari et al., 2012; Ghotbedin et al., 2013; Jalilifar et al., 2017a,b). Therefore, the present study aimed to investigate the time-dependent effects of LFS using kindling signal processing. In this regard, we comparatively evaluated the quantitative features of kindle group, and LFS-induced changes in two protocols of LFSK and KLFS with spectral assessments using FFT analysis.

Quantitative assessments of EEG signals demonstrated that administration of LFS reduced beta and gamma oscillations as well as increased delta sub band power. Moreover, alpha power decreased following application of LFS, but the reduction was not statistically significant (**Figures 5–7**). Application of LFS prior the kindling stimulation (LFSK) reduced beta and gamma sub bands power more than KLFS but the difference remained insignificant (**Figure 8**).

Since we aimed to quantitatively analyze EEG signals related to the Racine stages, we only focused on the part of the AD that occurred with emerging different seizure stages. Therefore, other parts of the AD which corresponded to other behavioral states were excluded from the analysis. According to the power spectrum figures (**Figures 5–7**), with progression of the kindling acquisition, larger peaks occurred in the mid (8–12 Hz) and high (12–40 Hz) frequencies and also a remarkable shift toward higher frequencies was evident at higher frequencies. However, there was no significant difference in the power spectrum figures between the LFSK and KLFS groups.

Our results showed no significant difference in the power of LFB, MFB, and HFB components in ISSs, LSS, and GSSs between the experimental groups. MFB and HFB components were considerably higher in the kindle group than the LFSK and KLFS animals, whereas LFB increased in both LFSK and KLFS groups (**Figure 6D**). Moreover, our findings demonstrated that high frequency components were increased at GSSs in all the experimental groups vis-a-vis LSS of the kindling (**Figures 6** and **7**).

We have analyzed the background EEG of the experimental groups for 7 s before the start of the kindling stimulation to clarify whether LFS only changed seizure activities or whether it also affected the background EEG. Since the results confirmed no significant differences in LFB, MFB, and HFB powers of the baseline EEG signals between the experimental groups, it is claimed that the group differences can only account for the seizure activity but not the background EEG. We previously reported increase of delta and theta oscillations with generalization of the kindling process (Jalilifar et al., 2016). In this regard, power spectrum results confirmed that the LFB (delta and theta sub-bands) is dominant with the progression of the kindling acquisition. We also found increase of HFB components in the GSSs. It can be observed that application of LFS especially in LSS and GSSs of the kindling acquisition reduced HFB components. Besides, more peaks occurred in the power spectrum figures of GSSs at higher frequencies as compared with LFS treated animals. In fact, there were larger peaks in GSSs of kindle animals at frequencies above 15 Hz whereas in the GSSs of the LFS treated group these picks were located at lower frequencies. To prove the above mentioned idea, application of LFS either before or after the kindling stimulation significantly increased delta power (**Figure 8A**). Moreover, the results showed that HFB power was increased in both LFSK and KLFS groups but the difference in the LFSK was only significant (**Figure 8**). Our behavioral data showed that ADD in the kindle group was significantly higher than the LFSK and KLFS (*p* < 0.05). However, to determine power spectrum figures and also EEG signal processing, we only focused on the part of AD that includes Racine stages and the parts of ADD related to other behavioral states were excluded from the signals. Therefore, we only quantitatively analyzed the duration of different stages of the kindling. Different studies have offered the ratio of theta/alpha as an important index for analyzing epileptic seizures (Fernández et al., 2003; Sadighi Alvandi et al., 2015). Our data indicated increase of theta/alpha ratio following the application of LFS. However, this increase would be much higher if LFS was applied before the kindling stimulation, compared with the application of LFS after the termination of kindling stimulation. The difference may be related to greater inhibitory effect of LFS when it was applied before the kindling stimulation than KLFS.

The results showed no significant correlation between each frequency sub band and ADD in different stages of Racine in the kindle group. There was a low negative correlation between ADD and MFB and also low positive correlation between ADD and LFB in ISSs in the kindle group. In addition, a weak correlation between ADD and different frequency sub bands was observed in the LFS treated animals.

The exact mechanisms of the antiepileptogenesis effects of LFS are not clearly determined, but two main theories have been proposed on the mechanism of action of LFS or more widely electromagnetic modulations on neural activities: dispersion theory and LTD. In the dispersion theory, the main idea is that injecting an external energy, usually in the form of electrical or electromagnetic fields can disturb or disperse the ongoing intrinsic neural activities. The impaired ongoing neural activities can be disturbed by an external electric field in appropriate frequencies.

Tsuchiya and Kogure (2011) found a strong positive correlation between an increase of high frequency components (12–30 Hz) and the behavioral progression of kindling, whereas decrement of these high frequency activities was associated with incomplete kindling stages which cannot support our findings (Tsuchiya and Kogure, 2011). Similar to our results, Musto et al. (2009) demonstrated that electrical kindling development could be suppressed by reducing gamma oscillations (21–40 Hz) indicating that the beta (12–20 Hz) and gamma waves in hippocampus were involved in the propagation of kindling seizures (Musto et al., 2009). Likewise, Dugladze et al. (2007) surveyed chemical kindling using kainic acid and they found suppressing theta waves meanwhile increasing gamma oscillations with behavioral progression of kindling (Dugladze et al., 2007). In this regard, application of LFS before the kindling stimulation, though not considerable, reduced beta and gamma power more than the KLFS group which confirmed more inhibitory effect of LFS when applied immediately before the kindling stimulation (**Figure 7B**).

Low frequency stimulation can also inhibit epileptogenesis process through LTD phenomenon (Goodman et al., 2005; Ozen et al., 2008). In this regard, application of LFS seems to suppress the release of glutamate and increase of inhibitory receptor activity and consequently leads to decrease of beta and gamma frequencies.

Furthermore, alteration in the glutaminergic neural activity following amygdala kindling may happen in CA1 neurons of hippocampus. In this regard, Ueda and Tsuru (1995) reported increase of the amount of NMDA receptors and release of glutamate during amygdala kindling which might be involved in generalization of kindling seizures (Ueda and Tsuru, 1995). Thus, reducing NMDA receptors can be another antiepileptogenesis mechanism of LFS and the reason for decrease of high frequency components (12–40 Hz). There are many reports that the disappearance of high frequency activities causes a relative increase of low frequency oscillations of the targeted neurons, which in turn inhibit the development of epileptogenesis. Delta and theta frequencies are implicated in maintenance of inhibitory system of hippocampus and amygdala regions (Miller et al., 1994). Moreover, increase of LFB contribution including delta band following application of LFS occurred due to decrease of the excitatory neural network activity (Dugladze et al., 2007).

Our findings showed that LFS immediately before or after kindling stimulations significantly inhibit kindling-induced epileptogenesis where the LFSK showed greater inhibiting effects than KLFS. It indicates that time of LFS application does not result in significantly different antiepileptic effects. However, some of the previous studies have reported the time of LFS application is an important factor in exerting the antiepileptic effects. In a similar study, Shahpari et al. (2012) compared the antiepileptic effects of LFS with the same protocol of our study immediately before kindling and 5 min after the termination of kindling and reported that LFS immediately before kindling stimulation induced more inhibiting effects (Shahpari et al., 2012). Considering the non-significant difference between LFSK and KLFS in antiepileptic effects, it seems that LFS partly exerts its inhibiting effects through the dispersion mechanism. This can be concluded that time of LFS application However, the difference resulted from the different studies conducted in two separate laboratories should be accounted. It can be concluded that a combination of wide dispersion and LTD might be the main responsible mechanism of action of LFS antiepileptic effects.

Administration of the same LFS protocol immediately before or after kindling stimulations significantly inhibit the kindling induced epileptogenesis in a way that LFS before the kindling stimulations exerts greater inhibiting effects than the KLFS protocol but the difference was not significant. Similarly, the quantitative measures including delta, beta and gamma oscillations, and theta/alpha ratio showed the greater changes in the LFSK than the KLFS group.

One of the main limitations of the present study is that the number of rats that reached the GSSs in the LFSK and KLFS groups was low. Investigating the effects of LFS alone on the baseline EEG signals of the amygdala could result in usefull information on determining markers for treatment response monitoring. Moreover, it is recommended to study the effects of kindling and LFS on the other regions in the brain to identify the associated measures and the spatial distribution of the effects. In our studies due to technical limits in the recording system we did not investigate these effects. Further studies are needed with bigger sample sizes and with more rats showing stages 4–5 to reach more reliable results.

## Conclusion

The LFS in both protocols can inhibit the kindling induced epileptogenesis. Our findings showed that application of LFS can increase the delta power while decreasing beta and gamma oscillations. It seems that LFS can provide antiepileptogenic effect through increasing LFB, meanwhile decreasing high frequency components which can be a helpful index for identifying LFS-induced antiepileptogenesis. Moreover, application of LFS before the kindling stimulation decreased beta and gamma oscillations and theta/alpha ratio more than the KLFS protocol which may be related to more inhibitory effects of the LFSK protocol compared with the KLFS. Behavioral assessments showed relatively the same level of antiepileptogenic effects for KLFS and LFSK, quantitative assessments showed more significant differences in the quantitative measures between the two protocols. Developing more quantitative EEG based measures correlated with LFS-induced effects can facilitate developing open or closed loop seizure prevention modalities.

## Author Contributions

MJ and AY contributed in study design and methodology, data collection and analyses, and manuscript preparation. AM and ZG contributed in the methodology and data collection and reviewed the manuscript.

## Conflict of Interest Statement

The authors declare that the research was conducted in the absence of any commercial or financial relationships that could be construed as a potential conflict of interest.
